# Crowdsourcing the identification of organisms: A case-study of iSpot

**DOI:** 10.3897/zookeys.480.8803

**Published:** 2015-02-02

**Authors:** Jonathan Silvertown, Martin Harvey, Richard Greenwood, Mike Dodd, Jon Rosewell, Tony Rebelo, Janice Ansine, Kevin McConway

**Affiliations:** 1 Department of Environment, Earth and Ecosystems, The Open University, Milton Keynes, MK7 6AA, UK; 2 Institute of Educational Technology, The Open University, Milton Keynes, MK7 6AA, UK; 3 Faculty of Maths, Computing and Technology, The Open University, Milton Keynes, MK7 6AA, UK; 4 South African National Biodiversity Institute, Kirstenbosch, Claremont, Cape Town, South Africa; 5 Current address: Institute of Evolutionary Biology, School of Biological Sciences, University of Edinburgh, Charlotte Auerbach Road, Edinburgh EH9 3FL, Scotland, UK

**Keywords:** Biodiversity, Citizen Science, Crowdsourcing, Identification, Learning, Learning design, social networking

## Abstract

Accurate species identification is fundamental to biodiversity science, but the natural history skills required for this are neglected in formal education at all levels. In this paper we describe how the web application ispotnature.org and its sister site ispot.org.za (collectively, “iSpot”) are helping to solve this problem by combining learning technology with crowdsourcing to connect beginners with experts. Over 94% of observations submitted to iSpot receive a determination. External checking of a sample of 3,287 iSpot records verified > 92% of them. To mid 2014, iSpot crowdsourced the identification of 30,000 taxa (>80% at species level) in > 390,000 observations with a global community numbering > 42,000 registered participants. More than half the observations on ispotnature.org were named within an hour of submission. iSpot uses a unique, 9-dimensional reputation system to motivate and reward participants and to verify determinations. Taxon-specific reputation points are earned when a participant proposes an identification that achieves agreement from other participants, weighted by the agreers’ own reputation scores for the taxon. This system is able to discriminate effectively between competing determinations when two or more are proposed for the same observation. In 57% of such cases the reputation system improved the accuracy of the determination, while in the remainder it either improved precision (e.g. by adding a species name to a genus) or revealed false precision, for example where a determination to species level was not supported by the available evidence. We propose that the success of iSpot arises from the structure of its social network that efficiently connects beginners and experts, overcoming the social as well as geographic barriers that normally separate the two.

## Introduction

All fields of biodiversity science depend upon the fundamental requirement that the species under study must be correctly identified. Although biodiversity science has experienced explosive growth due to the advent of sophisticated tools such as next generation sequencing that can characterise microbiomes containing thousands of operational taxonomic units (Castelle et al. 2013) or satellite remote sensing that can monitor ecosystems across the entire globe (Buchanan et al. 2009), field identification skills have languished. The natural history knowledge required to correctly identify species in the field is now rarely taught in biological curricula in schools or universities, either in Europe or North America (Tewksbury et al. 2014). In this paper we demonstrate that learning technology combined with crowdsourcing that connects beginners with experts can make an important contribution to solving this problem.

Crowdsourcing is the practice of seeking the execution of a task or the solution to a problem through an open invitation for anyone to participate (Doan et al. 2011). It is often used in citizen science, for example in the many projects hosted by Galaxy Zoo, to process large volumes of data (Nielsen 2012). In its turn, citizen science – the practice of science by volunteers with or without direction by professional scientists (Silvertown 2009) – has a long tradition in the field of biodiversity research where observations by amateurs, many of whom are experts in their own right (Ellis and Waterton 2005), constitute the bulk of the data that exist, for example on the distribution patterns of species (Bell et al. 2008; Mackechnie et al. 2011; Preston 2013; Roy et al. 2014). In this paper we describe the design and operation of a crowdsourcing web application, ispotnature.org and its sister site ispot.org.za (collectively, “iSpot” and now merged), that we have created to help anyone, including citizen scientists, students and naturalists, with species identification. While there are other websites such as iNaturalist.org, ProjectNoah.org, Discoverlife.org and Bugguide.net that crowdsource the identification of organisms, we are unable to make a comparison with them because they have not yet been the subject of published analysis.

iSpot crowdsources the identification of organisms shown in photographs submitted by participants to an online social network designed and dedicated to the purpose. Facebook, Twitter and Flickr are all widely used for identification (Barve 2014; Deng et al. 2014), but these social networks lack the specific functions required to verify organism names. It is arguable that observations that lack a verified identification are of limited value to science and are a doubtful basis for learning. iSpot also allows participants to build taxon-specific reputation and it links observations to each other *via* their taxonomic and ecological relationships. These functions are used to build additional features such as quizzes that assist with verification and learning. By focussing on learning, iSpot not only helps participants generate valid scientific observations, but it also trains them to become the biological recorders on whom future data collection will depend.

## Social network structure

**Figure 1.**
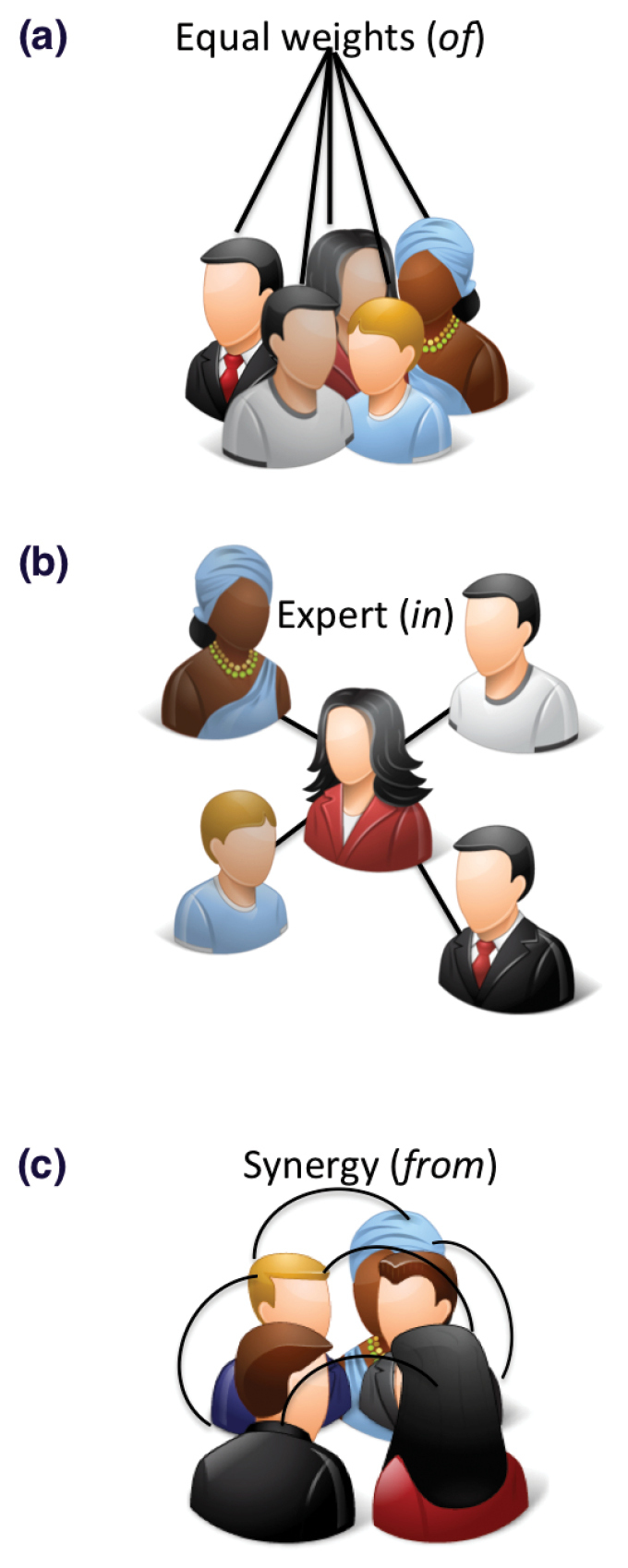
Schematic of three network structures, from equal status, to expert status and a hybrid system. (Icons http://www.icons-land.com)

iSpot is explicitly designed around a social network structure suited to verifiable identification and learning. Three different types of social network structure are schematically represented in Figure 1. In the network shown in Fig. 1a, the contributions of all participants have equal weight. The result might be described as sourcing the knowledge *of* the crowd or *unstructured crowdsourcing*. This is suited to tasks where all participants are equally qualified by virtue of the task being very simple or the ‘crowd’ being carefully selected to contain only experts, but it is otherwise unsuited to the identification of any but the most common organisms. The social structures of Facebook and Twitter correspond to this kind of model.

Fig. 1b represents a social network in which a recognised expert is the source of identification. Here, the function of the crowd is simply to increase the size of the social network to the point at which experts, who are necessarily rare individuals, are statistically likely to be included. We characterise this as sourcing the knowledge *in* the crowd. This network structure is clearly superior to the first one in obtaining correct names, but it requires a means of verifying expertise and of directing experts’ attention to relevant observations.

The third type of social network structure (Fig. 1c) represents obtaining knowledge *from* the crowd. This network is synergistic, producing a result that cannot be obtained just from the sum of its parts. iSpot has deliberately been designed to have a hybrid structure that has features of both (b) and (c). The reason we chose this is because while (b) is an efficient, and indeed the traditionally accepted way to obtained verified names, it does not allow beginners to be involved in the process of identification and therefore it is unsuited to learning.

**Figure 2.**
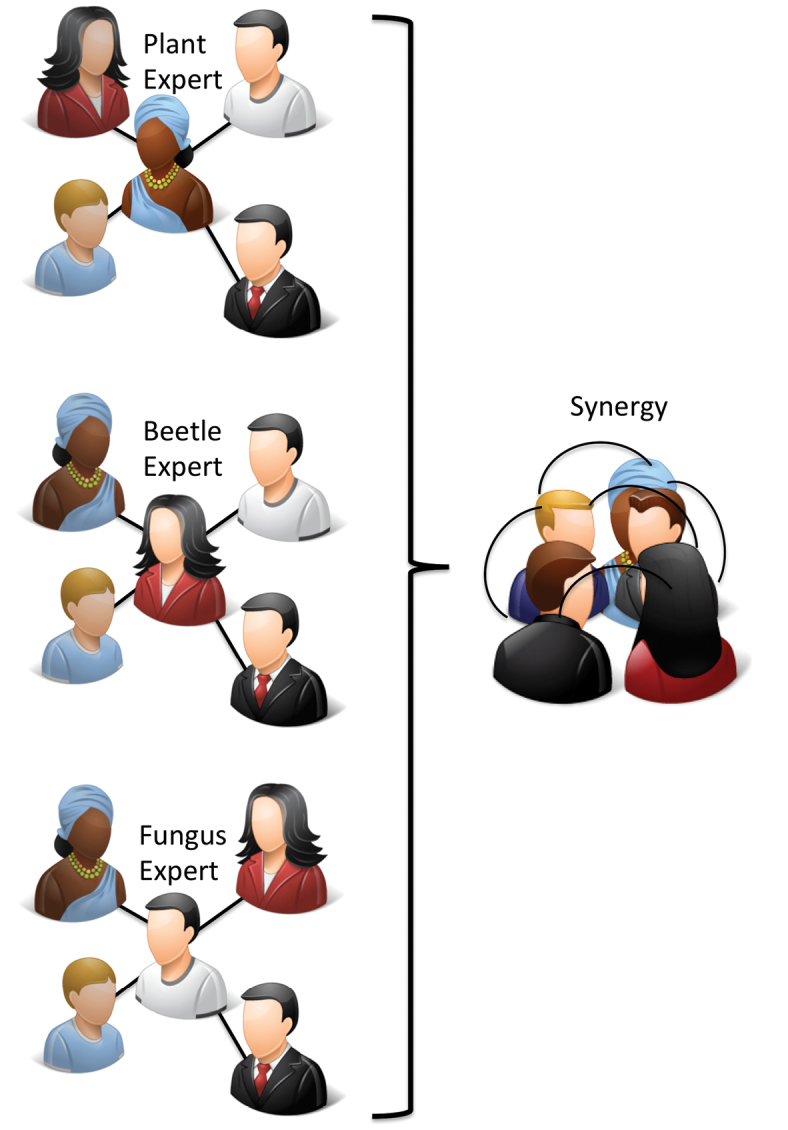
The conceptual social network structure of iSpot, showing the group (3 of the 8) compartmentalization and its interaction. Not shown is the learner-mentor interaction within each group. (Icons http://www.icons-land.com)

The hybrid social network structure of iSpot shown schematically in Fig. 2 takes advantage of the fact that no one can be an expert in the identification of all taxonomic groups and therefore, in a social network that covers all taxa, everyone is a beginner in at least some domain of knowledge represented on the website. The result is a social network that is friendly to beginners, even though it contains many experts who are highly knowledgeable. This structure also allows participants to conduct a learning journey from beginner to intermediate and expert status, while contributing to the core identification purpose of iSpot as they do so.

**Figure 3.**
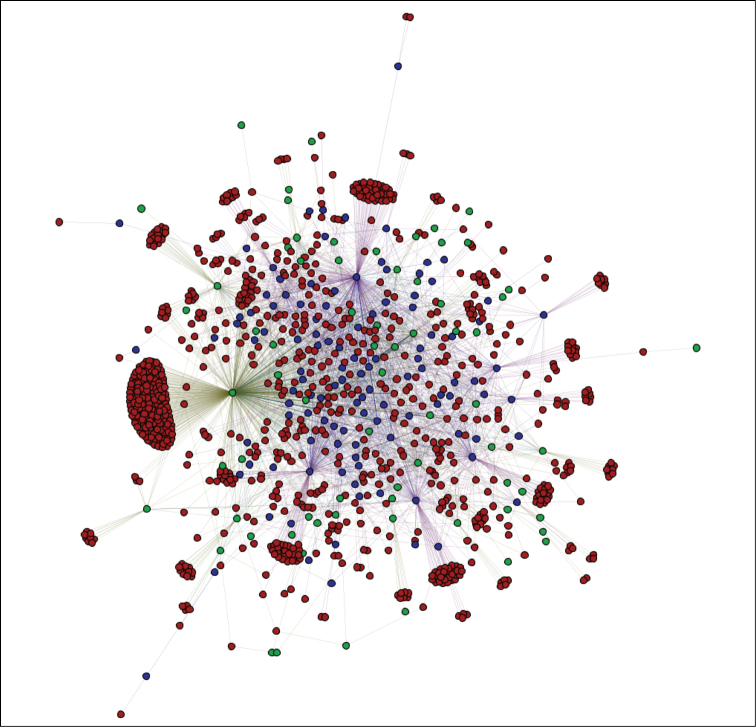
The network linking participants who posted observations to iSpotnature.org without an identification and those providing a likely identification for those observations. The diagram is based on the sample of 5,000 identifications made up until 1 July 2014. This activity occurred over 32 days. The network contains 1,110 nodes linked by 2,876 edges. Red nodes (83.23%) are participants who only received identifications, green nodes (6.38%) are participants who only made identifications and blue nodes (10.39%) are participants who both made and received identifications.

Formal network analysis of iSpot will be the subject of a future publication, but a preliminary insight into the iSpot social network can be obtained Fig. 3 which shows recent interactions in iSpot. An edge shows where one participant provided a likely identification to an observation made by another participant. Red nodes are participants who, in this period, only received determinations from others; green nodes are participants who only provided determinations to others. The hybrid nature of the network is suggested by the blue nodes – participants who both made and received determinations – which include ‘hub’ individuals who are some of the most active participants in the network.

**Figure 4.**
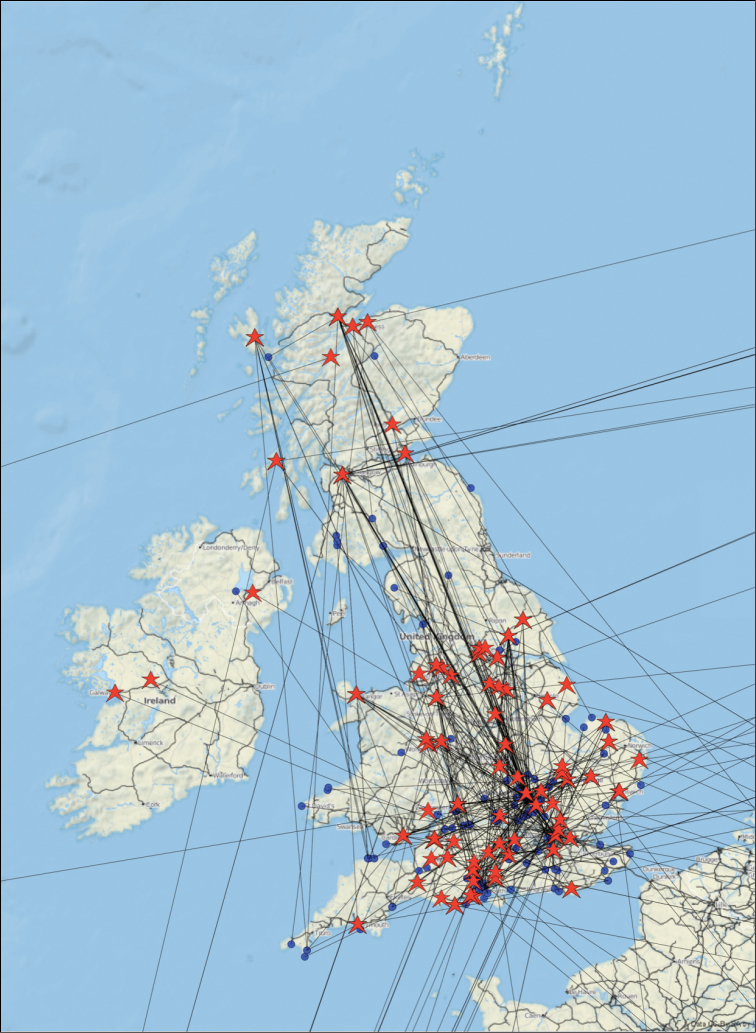
A map of the social network of a single participant who contributed about 500 observations to iSpotnature.org at the locations shown by blue dots, with other iSpot participants, living at locations shown by red stars, who provided determinations for those observations. The location of the observation and the location of the person identifying it are joined by a line.

A different perspective on the social network is provided by the map shown in Fig. 4 of the geographic location of > 500 observations made by one much-travelled participant of iSpot. This spatial realization of the social network of identifiers who determined the observations of just one iSpot participant illustrates how iSpot successfully connects people who need identification assistance with the multitude of those who are able and willing to help them.

## The iSpot reputation system

The threefold requirement to identify experts, verify identifications and involve beginners in the social network is achieved through iSpot’s purpose-designed reputation system. There is a large and diffuse literature about systems for establishing reputation and trust in online social networks (Josang et al. 2007; Scekic et al. 2013), but most of this concerns commercial transactions on websites such as eBay (Beldad et al. 2010), or deals with methods of computing reputation indirectly from network structure (Jiang et al. 2014).

In the specific instance of designing a reputation system for online identification of organisms we have two advantages that do not apply in e-commerce. Firstly, high reputation in the online social network can be pre-assigned (seeded) to designated individuals who have already earned expert status in identification in an offline context. Secondly, social interactions in iSpot are mediated by a social object, the observation, and are not purely dependent on social relationships (Clow and Makriyannis 2011). Thus, in iSpot we have two exterior reference points that help calibrate online reputation, namely: 1. Whether participant X is a recognised expert in their field, and 2. Whether such experts agree on the identity of observation Y. Both of these reference points are of course themselves social constructs because they depend, in the first instance on *recognition*, and in the second on *agreement*, but the system is robust because, (i) only a very small fraction of participants are pre-assigned a reputation rather than earning it and (ii) the iSpot reputation system continually tests all participants’ online identifications so that everyone, including those designated (pre-assigned) “knowledgeable”, have an earned reputation score that is visible in their profile.

**Figure 5.**
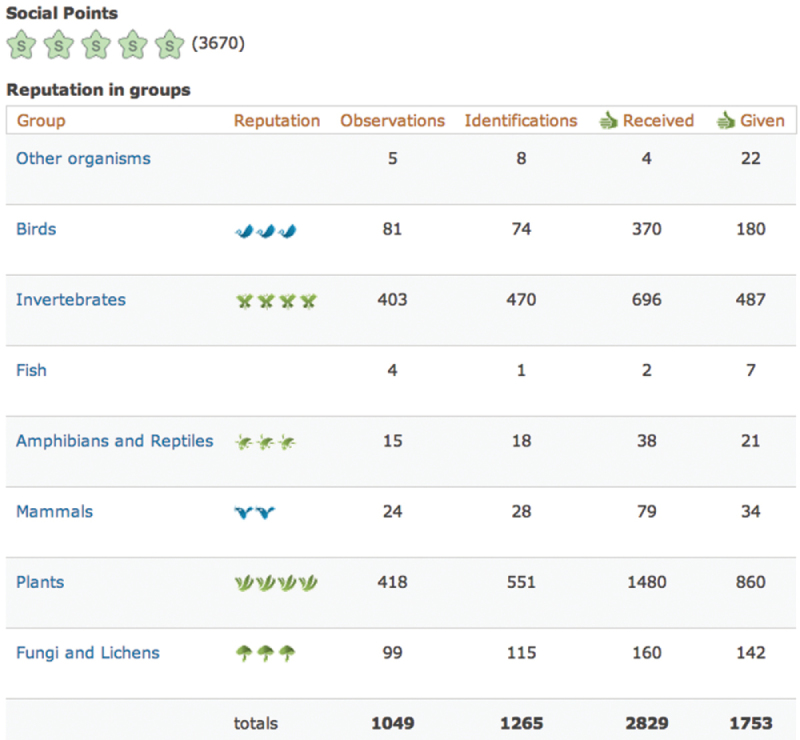
An example of an iSpot participant’s profile showing their reputation and activity in the 8 groups.

The iSpot reputation system works as follows. Each participant has a 9-dimensional reputation (Fig. 5). One of the dimensions is the participant’s social reputation which simply records the amount of their activity on the site, doing things such as posting comments or offering identifications on other people’s observations. The remaining 8 dimensions are reputation scores, based upon the weighted agreements given by other participants, for identifications made in the 8 groups: birds, fish, herptiles (amphibians and reptiles), invertebrates, mammals, plants, fungi and lichens and ‘other organisms’. This set of groups is pragmatic rather than strictly taxonomic, but it has the merit of being simple enough to allow participants of all abilities to classify their observations in a useful way, even if they cannot identify an organism any more precisely. We discuss the limitations of using only 8 groups for reputation purposes later.

iSpot attaches badges to usernames when displayed on screen to signal a participant’s reputation, with a different icon for each of the 8 groups. For example a stylised bird indicates bird reputation, a butterfly indicates invertebrate reputation and so on. A participant may have up to five icons in any group, with the number of icons awarded being scaled geometrically with the numerical reputation score that has been earned. Only a low score is required to earn one bird icon, but a very high cumulative score is required to earn five. A participant’s full, 9-dimensional reputation is visible on their profile page (Fig. 5), but on all other pages iSpot displays only the reputation badge that is relevant in the context of that page. So, on a page for a bird observation, only badges representing participants’ bird reputations are shown.

When an iSpot participant posts an observation they are required to select which of the 8 groups it belongs to and they can also add the name of the organism if they think they know it. In order to encourage participants to utilize and hence test whatever level of knowledge they may have, iSpot allows a choice of three levels of confidence when a name is proposed. Where we have them, region-specific dictionaries of scientific and vernacular names are used to check that proferred names are correctly spelled and are current. Where we do not have a local dictionary, we use the global Catalogue of Life (“Catalogue of Life http://www.catalogueoflife.org/”). A participant who is suggesting a name must select between: “I’m as sure as I can be”, “It’s likely to be this but I can’t be certain” and “It might be this”.

**Figure 6.**
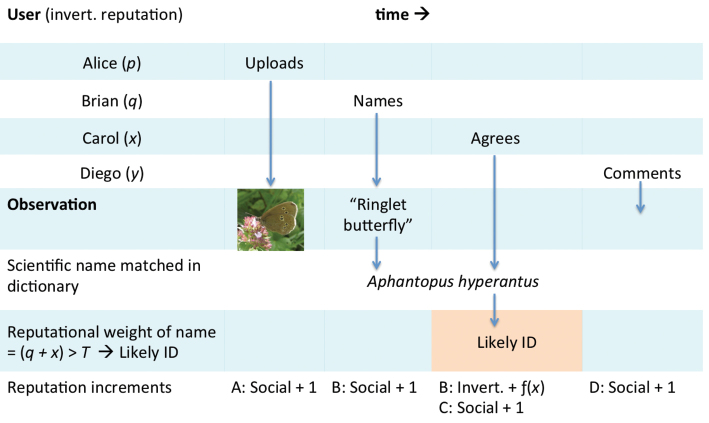
Schematic showing how the iSpot reputation system works. See the text for further explanation.

The operation of the reputation system is illustrated in Fig. 6. In this example an observer called Alice uploads a photograph of a butterfly that she would like identified. Brian thinks it is a Ringlet and iSpot matches this vernacular name to the binomial *Aphantopus hyperantus* using the species dictionary relevant for the location in which the observation was made. Carol sees this observation and agrees with the name that Brian has suggested. iSpot calculates the total reputational weight attached to this name as the sum of Brian and Carol’s reputations in invertebrates (*q* + *x*). If this total exceeds a pre-set threshold value *T*, iSpot marks Ringlet, *Aphantopus hyperantus* as the Likely ID. If more than one name is proposed and both have a reputational weight > *T*, iSpot marks the one with the highest value as the Likely ID, or where these are equal the latest name. In this example, Brian made the determination and so his reputation for invertebrates is increased by an amount that is scaled by Carol’s invertebrate reputation. The maximum increment Brian can earn is 1. This will happen if Carol is an expert, or if several people with lower reputational scores that sum to unity or more all agree with Brian’s ID. All actions except agreeing shown in the diagram earn participants one social point. Social points are not used to determine Likely ID status.

We have evaluated the characteristics of the iSpot system against the reference model for online reputation systems proposed by Vavilis et al. (Vavilis et al. 2014) in Table 1. Vavilis et al. compared 15 different reputation systems using their model and found that none satisfied all the requirements fully. iSpot comes close to doing this, but contravenes R9 “Users should not gain advantage of their new status.” This particular requirement is not appropriate in a system designed for learning because it is desirable to encourage new participants by making it easier for them to gain reputation.

**Table 1.** The characteristics of the iSpot reputation system evaluated against the requirements for online reputation systems proposed by Vavilis et al. (Vavilis et al. 2014). The characteristics shown apply to the 8-dimensions (groups) of the taxonomic reputation, not social reputation scores.

ID	Requirement	iSpot reputation system
R1	Ratings should discriminate user behaviour.	Ratings are algorithmically awarded based upon weighted agreements and discriminate between user activity in different taxa (groups).
R2	Reputation should discriminate user behaviour.	Reputation is a scaled function of ratings and hence discriminates user behaviour.
R3	The reputation system should be able to discriminate “incorrect” ratings.	The reputation system only awards a Likely ID when its threshold is crossed and it selects better supported over less well supported names when more than one is proposed.
R4	An entity should not be able to provide rating for itself.	Users proposing a name cannot ‘agree’ with themselves.
R5	Aggregation of ratings should be meaningful.	Multiple agreements are the norm and represent the ratings of different users. There is no incentive for gratuitous agreement.
R6	Reputation should be assessed using a sufficient amount of information.	Reputation is earned from all agreements and is cumulative within groups.
R7	The reputation system should differentiate reputation information by the interaction it represents.	The 8 dimensions of the reputation system differentiate users by their taxonomic field of expertise. Social points are earned and aggregated separately from ID-based reputations.
R8	Reputation should capture the evolution of user behavior.	Reputation, earned by contributing correct identifications is dynamic, but it can only increase.
R9	Users should not gain advantage of their new status.	This requirement is contrary to the learning principle utilized in iSpot, which is that new users need to be encouraged with early rewards. In iSpot, it is easier to earn the first one or two reputation badges in a taxon than later ones. However, although it is easier to gain early status, relative ranks of users can only be changed by making valued contributions.
R10	New users should not be penalized for their status.	New users are encouraged (See R9).
R11	Users should not be able to directly modify ratings.	Users cannot directly modify their ratings.
R12	Users should not be able to directly modify reputation values.	Users cannot directly modify their reputation.
R13	Users should not be responsible for directly calculating their own reputation	Reputation is calculated algorithmically, not by users themselves.

The Vavilis et al. model does not rate reputation systems directly according to how easy they are for participants to game, although defence against gaming is an important requirement. The iSpot system is quite resistant to gaming for a number of reasons. Friedman et al. (2007) consider three general strategies that are used by reputation cheats: whitewashing, dishonest feedback and sybil attacks. In whitewashing, a participant abandons an identity that has low reputation and bad feedback and opens a new account. An iSpot participant who did this would effectively be re-setting their reputation to zero rather than improving it, so there would be no point in doing it. In the context of iSpot, “feedback” is represented by giving agreements, but participants get no reputation or social points for giving agreement, so there is no incentive to do this falsely.

A sybil attack is when a participant enhances their reputation by creating fake accounts that give their primary account positive feedback. Here too, there is no real incentive in iSpot for a participant to do this. The fake accounts would start with zero reputation and their influence on the Likely IDs won by the primary account would therefore be negligible. High taxonomic reputation can only be earned by consistently proposing names that attract the agreement of highly-ranked participants.

It is possible for a participant to submit fake, duplicate or ‘stolen’ observations, but reputation would only be earned from these if they were correctly named and agreed-with. In practice we have not seen this happen, probably because such behaviour would quickly become apparent to other iSpot participants and would therefore be self-defeating.

## The performance of iSpot

### Numbers of participants, observations and taxa identified

The two iSpot websites between them received more than 2.2 million visits from nearly 900,000 unique visitors (Table 2).

**Table 2.** Cumulative usage statistics up until 30 June 2014 for the two iSpot platforms www.ispotnature.org (mainly UK observations, launched June 2009) and iSpot Southern Africa www.ispot.org.za (launched June 2012). Data available from the Dryad Digital Repository: http://doi.org/10.5061/dryad.r0005

Number of	UK	%	ZA	%	Total	%
Unique visitors	724,272	-	166,936*	-	891,208	-
Participants registered	35,988	-	6,455	-	42,443	-
Observations uploaded	262,942	-	127,222	-	390,164	-
Determinations made	322,076	-	159,675	-	481,751	-
Agreements given	1,020,539	-	241,431	-	1,261,970	-
Images uploaded	423,501	-	287,776	-	711,277	-
Registered participants adding an observation	13,644	37.65%	1,895	29%	15,539	37%
Registered participants adding a determination	8,408	23.20%	1,555	24%	9,963	23%
Registered participants adding an agreement	5,870	16.20%	1,087	17%	6,957	16%

* Since June 2013 only.

**Figure 7.**
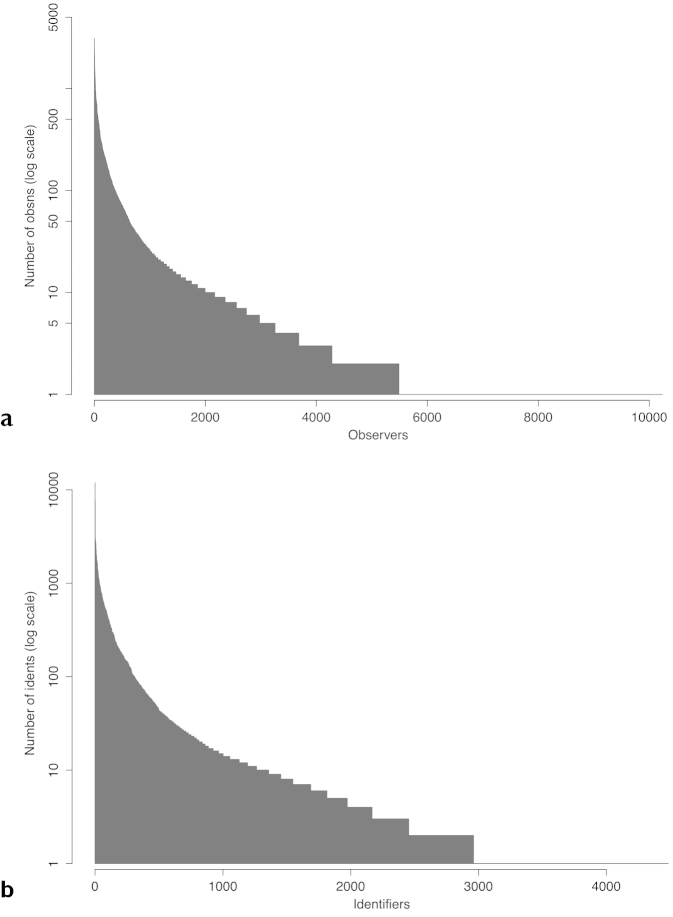
**a** iSpot participants who made at least one observation, ranked on the horizontal axis by the number of observations each made (shown on the vertical axis). **b** iSpot participants who made at least one determination ranked on the horizontal axis by the number of identifications each made. n = 201,711 observations on ispotnature.org for both.

As at June 30th 2014, iSpot had a global total of more than 42,000 participants (Table 2). Fewer than half of registered participants added an observation, just under a quarter added a determination and only 16% registered an agreement with a determination. While tens of thousands of participants contributed to the 390,000 observations recorded, the majority contributed just a handful of observations each, while a few hundred power-users contributed hundreds or thousands of observations each (Fig. 7a). As might be expected, the frequency distribution of determinations per participant is even more skewed towards contributions from a minority of very active participants (Fig. 7b). This pattern of participation inequality has been observered in other citizen science projects (Nov et al. 2011).

**Figure 8.**
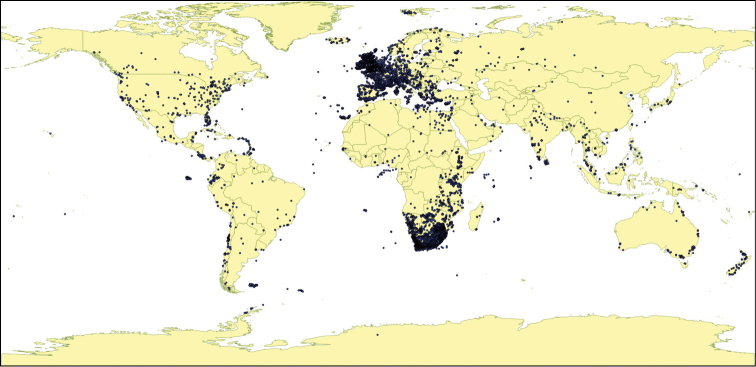
The global distribution of observations made on ispotnature.org and ispot.org.za up to December 2013.

The majority of observations (85%) submitted to ispotnature.org came from the UK, but the remainder were observed in 138 other countries or territories (Fig. 8).

### Frequency and speed of determination

**Figure 9.**
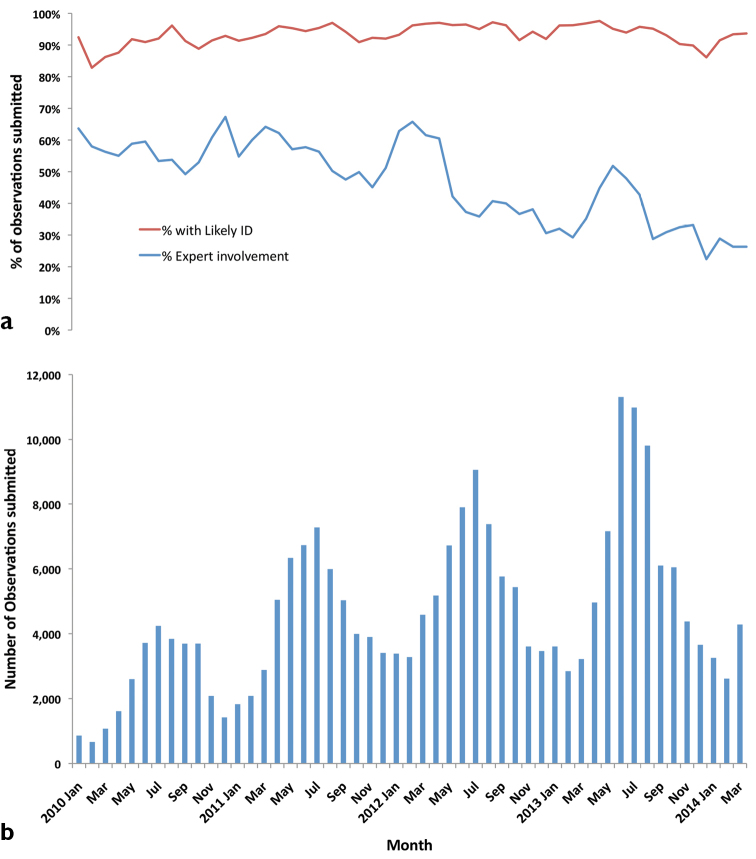
For ispotnature.org in the 4-year period shown: **a** The percentage of observations submitted each month that received a likely ID and the percentage where an expert provided or agreed the determination **b** The number of observations submitted per month.

How well does iSpot perform its core function of attaching verified names (determinations) to observations and how scaleable is this? Fig. 9 shows the answers to these questions for all 234,000 observations submitted to ispotanature.org up to the end of March 2014. Likely IDs were acquired by more than 90% of the submissions made each month and the overall determination frequency during the period of 4.25 years was 94% (Fig. 9a). The large majority of determinations (80%), were to species level (see below).

The bar chart in Fig. 9b shows how the number of submissions per month varied with season and grew year-on-year. Figs 9a and b together demonstrate that the determination rate in iSpot is scaleable and robust to an increase of more than 132% in the number of observations submitted. In fact, the determination frequency even improved slightly from 92% (n = 36,293) in the first 15 months to 94% (n = 84,232) in the last 15 months of the time series. Fig. 9a also shows that the percentage of observations in which a designated expert either made the determination or agreed with it declined very markedly over the period, but without reducing the determination frequency.

Of 109, 447 observations submitted to ispot.org.za, 95% received a name. Across all taxa, 83% were determined at species level, but this percentage varied from a high of 97% for mammals (n = 3,242) and 98% for herptiles (n = 5,513) to only 52% for fungi & lichens (n = 1,961) and 45% for insects (n = 15,590). The remaining determinations in the latter groups were made at the levels of genus, family or order.

To obtain an independent check on the names acquired by iSpot observations we submitted 46,736 observations of plants and moths made in the UK to the biological recording website iRecord (http://www.brc.ac.uk/irecord) run by the UK Biological Records Centre. Because of the volume of records submitted, only about 8% of them, a sample of 2,234 plants and 1,053 moths, were examined by experts in iRecord, but of these 94% of the plant records and 92% of the moths were verified.

**Figure 10.**
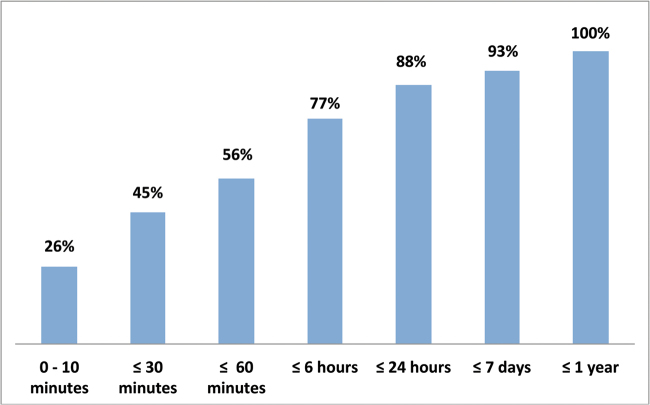
The cumulative frequency distribution for the time taken for a likely ID to be acquired by observations submitted to ispotnature.org without an organism name. n = 100,703 observations.

iSpot is not only efficient in providing verified names, but it is also fast. Over half the UK observations submitted without a name were determined with one hour and 88% of such observations acquired a likely ID within 24 hours (Fig. 10). With a smaller participant community and a much more diverse biota, identification was slower on ispot.org.za, but the community still identified 50% of observations within 10 hours of submission.

### Accuracy and precision of Likely IDs

iSpot relies on photographs for identification. How accurate and precise are the determinations made this way? To answer this question we analysed in greater detail the 156,491 British observations made during the three years 2011–2013 that received at least one identification using a name that is present in the UK Species Inventory (UKSI) (“UK Species Inventory” 2014). Likely ID status is the only measure we have of accuracy, but precision can be represented by the taxonomic rank at which the Likely ID was made. There were 10, 672 taxa recorded in the whole sample, 80% at species rank, 14.5% at genus, 4% at family, 0.7% at order and 0.2% at phylum rank.

In 85% of the sample (132,835 observations), the likely ID was the only name submitted. Among the remaining 15% of observations (n = 23,606), the majority (21,613), comprising 13.81% of the whole sample, had two UKSI names proposed. Three names were proposed in 1,846 cases, four names in 135 cases and 12 observations had five names. All proposed names remain visible on iSpot and cannot be removed by the proposer. This preserves the discourse that takes place around contentious observations, providing a useful demonstration, especially for beginners, of how names may be arrived at.

Taking just the sample of 14,611 observations where there were two names and the alternative name as well as the Likely ID were present in the UKSI, we were able to calculate improvements to the accuracy and precision of the Likely ID compared with the offered alternative. An increase in accuracy without a change in precision occurred when the Likely ID and its alternative had the same taxonomic rank, for example when *Pieris napi* the Green-veined White butterfly was the likely ID and the alternative name suggested was another butterfly in the same genus, *Pieris rapae*, the Small White. This particular confusion occurred 38 times out of 251 *Pieris rapae* IDs, while the reverse mistake occurred only 3 times out of 296 *Pieris napi* IDs. In other words, though the two butterflies were reported with similar frequency, the Green-veined white was mistaken for the Small White about ten times as often as the reverse. The other common British species in this genus is the Large white, *Pieris brassicae* (228 IDs) which was mistaken for *Pieris rapae* 15 times, but for *Pieris napi* only twice.

In the two-name sample as a whole (Table 3), the Likely ID provided a different name at the same rank as the alternative more than half of the time (57% of cases, Table 4). Most of these increases in accuracy (76%) occurred within the rank of species, followed by genus (12%) and family (8%).

**Table 3.** Taxonomic ranks of Likely IDs and of alternative names for a sample of 14,611 observations of UK species submitted to iSpot.

	Rank of alternative name						
Rank of Likely ID	Phylum	Class	Order	Family	Genus	Species	Total
Phylum	1	1	-	2	1	8	13
Class	2	3	1	6	12	51	75
Order	-	5	14	29	40	118	206
Family	4	10	68	86	128	441	737
Genus	4	18	118	200	295	1,807	2,442
Species	26	113	388	737	1,985	7,889	11,138
Total	37	150	589	1,060	2,461	10,314	14,611

**Table 4.** Percentage of changes in taxonomic rank for the data shown in Table 3, with the inferred results for precision and accuracy.

	Initial taxonomic rank						
Change in taxonomic rank (Inference)	Phylum	Class	Order	Family	Genus	Species	All ranks
Up (False precision in the alternative name)	-	1%	0%	3%	7%	24%	18%
None (Improved accuracy in the Likely ID)	3%	2%	2%	8%	12%	76%	57%
Down (Improved precision in the Likely ID)	97%	97%	97%	88%	81%	-	25%

We defined a change in the precision with which a Likely ID was made by comparing its rank to the rank of its alternative. An increase in precision occured when the rank of the Likely ID was lower than the rank of its alternative. For example when the Likely ID was *Bombus lucorum* and the alternative was *Bombus* sp. Improvements in precision from genus to species occurred in 81% of cases (Table 4).

The reverse relationship of ranks, with the rank of the Likely ID higher than the alternative, indicated that the alternative determination had been made with false precision. For example, there were 11 cases where the name *Bibio marci* (St. Mark’s fly) was proposed, but where the Likely ID was *Bibio* sp. because the precise species of the genus *Bibio* could not be determined. In the whole dataset, 18% of comparisons were of this type (Table 4).

## Discussion

Commentators on the progress of science tend to emphasize the impact of technological advances in hardware and with good reason. Particle accelerators, genome sequencers, satellites, MRI scanners and fast computers, to name just a handful of obvious examples, have been revolutionary. However, as Nielsen has argued in *Reinventing Discovery* (Nielsen 2012), there is another revolution also taking place in how science is done in a connected world. iSpot seeks to engineer social connections in the three-fold interests of biodiversity science, learning and conservation. That anyone can now get an identification of almost any organism within minutes or hours, entirely free, is potentially revolutionary. Three examples among many deserve to be mentioned. In Britain, a 6 year old girl discovered an unusual moth that was rapidly identified on iSpot as a species not seen in Europe before (iSpot 2009). In South Africa, a doctor submitted a photograph of unknown seeds that were the cause of poisoning in several children presenting at a clinic and these were identified 35 seconds after posting on iSpot (iSpot 2013). Hitherto unknown populations of South African endemic plant species are regularly discovered on iSpot.

The results reported here are the fruit of six years of community-building using both online and face-to-face methods in the UK (Ansine 2013) and in South Africa. More than 150 natural history societies are now affiliated with iSpot and new iSpot communities with local curators have recently been established in Chile and Hong Kong.

iSpot is not the first or the biggest source of identification on the internet, but we believe that this is the first report of how effective the process can be in a social network that has been specifically designed for the purpose. As such, iSpot is not only a source of identification and learning, but also a laboratory in which we can discover how to improve both. For example, there is valuable information in the mistakes that people make. In our analysis of accuracy and precision, we found that the mistakes made among the 3 common *Pieris* butterflies were not symmetrical, suggesting that lack of familiarity with all the species, particularly *Pieris napi*, rather than their similarity was the source of the difficulty that naive participants had in identifying these species. This kind of quantitative information will be used in future to improve the identfication tools available in iSpot. Although the sample size we analysed here was large (n = 23,606), it covered only a small proportion (15%) of observations and therefore many taxa of interest will have been omitted. A recently introduced feature is the iSpot quiz which draws content from our entire database of observations. In future we will be able to use data derived from mistakes that people make in the quiz to extend our analysis of identification errors to a much higher proportion of species.

While we have anecdotal evidence from comments made by participants that they have learned, we do not have direct, quantitative evidence of learning in iSpot yet. However, we do know from a previous analysis of 400 participants’ behaviour that they provided determinations for fewer than 40% of their very first observations, but that they themselves determined more than 60% of their 50th observations (Scanlon et al. 2014). This change in behaviour probably reflects learning, although other causes of the trend are possible.

We also have evidence that the effective operation of iSpot is becoming less dependent upon the limited number of experts whom we seeded with high reputations at the start. In January 2010, designated experts were contributing to 60% of determinations, but four years later 60% of observations were being determined without their input (Fig. 9). Fig. 9 also shows that there were clear peaks in the expert participation rate in advance of the seasonal peak in submissions in 2012 and coincident with the seasonal submission peak in 2013. This pattern may suggest that designated experts were trying and failing to keep up with the increasing volume of observations being submitted. The fact that the determination rate was not affected by this shows that participants with earned reputation were playing an increasingly more important role in iSpot over time. We interpret this as evidence that iSpotters were using what they had learned on iSpot to help each other identify their observations.

The iSpot reputation system achieves its three goals of identifying expertise, verifying identifications of organisms and involving beginners. It is resistant to gaming and performs well against generic requirements (Table 1), but it does have limitations. Just eight groups are used for taxonomic reputation and this low granularity means that reputation badges in, say invertebrates, do not reflect the very different domains of expertise of a participant who is expert in beetles compared to one who is expert in starfish. Neither does it discriminate between two participants who have both earned the same plant reputation, when one has done so by identifying plants in South Africa where there are 22,000 species and the other by using iSpot in the UK where there are fewer than 2,000. Given these obvious limitations, it is perhaps surprising that the iSpot reputation system works as well as it does. We suggest that there are three reasons why this is so.

Firstly, although all agreements are used in computing Likely IDs, those contributed by highly ranked and expert participants have much greater weight than those given by beginners. This makes the system robust to mistakes by beginners who will be the greatest source of errors, while still allowing participants of all abilities to contribute and to be involved. Secondly, higher-ranked and expert participants tend to guard their reputation by exercising caution, so while their agreements carry considerable weight they are also less prone to error. Higher ranked and expert participants are more aware of alternative taxa and problematic groups and tend to retreat to higher level identifications, thus providing the most appropriate degree of taxonomic precision and preserving the kudos in their reputation (though in fact iSpot reputation itself cannot be lost). iSpot also facilitates caution by attaching three levels of confidence when names are proposed and by providing a comments thread in every observation where experts can explain in detail why an organism cannot be *x*, why it could be *y* or *z* and how to tell *y* and *z* apart. As soon as an observation acquires a name, iSpot displays thumbnails of other observations of the species in a carousel on the page. This provides a visual check, so that misidentified observations are often immediately apparent. Participants quickly learn to both check for a match with the other observations and to be more cautious.

Thirdly, we suggest that the low granularity of the reputation classification in taxonomic and geographical space is supplemented by additional, hidden granularity engendered by the small-world properties of the social network. In a small-world network, people who are unknown to one another are linked by mutual acquaintances and the distance between any two nodes scales as the log of the number of nodes (Newman 2010). In iSpot, this would mean that regular participants with similar interests and similar location encounter each other repeatedly online. The social network in which the reputation system operates may therefore make it much more granular in practice than the use of just 8 taxonomic categories would imply. Another factor is time constraints and large numbers of observations, so that experts in any group tend to limit their contribution to their specialist taxa within the iSpot groups, and tend not to contribute much to other taxa, effectively increasing the granularity of the groups to a far finer degree than is apparent.

We have concluded that the strength of the iSpot reputation system lies in its combination of apparent simplicity with hidden complexity. It follows from this that the way to improve the system is not to make it more complicated for the user by increasing the number of dimensions of reputation, but to make the underlying source of a participant’s reputation more visible. We are working on intuitive ways of doing this.

Finally, we should mention that although we did not initially set out to create a reference database for species identification, that is increasingly what iSpot provides. We know from feedback that visitors, including environmental impact assessors, conservation officials, horticulturists, landscapers and even expert taxonomists, regularly use iSpot to check identifications. As iSpot continues to expand with new communities around the world, we expect to teach, to learn and to discover more.
